# Targeted delivery of CD40L promotes restricted activation of antigen-presenting cells and induction of cancer cell death

**DOI:** 10.1186/1476-4598-13-85

**Published:** 2014-04-17

**Authors:** Kim L Brunekreeft, Corinna Strohm, Marloes J Gooden, Anna A Rybczynska, Hans W Nijman, Götz U Grigoleit, Wijnand Helfrich, Edwin Bremer, Daniela Siegmund, Harald Wajant, Marco de Bruyn

**Affiliations:** 1 Department of Obstetrics and Gynecology, University of Groningen, University Medical Center Groningen, Groningen, The Netherlands; 2 Department of Molecular Internal Medicine, Medical Clinic and Polyclinic II, University of Würzburg, Würzburg, Germany; 3 Department of Nuclear Medicine and Molecular Imaging, University of Groningen, University Medical Center Groningen, Groningen, The Netherlands; 4 Department of Surgery, Surgical Research Laboratory, University of Groningen, University Medical Center Groningen, Groningen, The Netherlands; 5 Department of Hematology, Medical Clinic and Polyclinic II, University of Würzburg, Würzburg, Germany

**Keywords:** CD20, EpCAM, CD40L, ScFv, Targeting, Fusion protein

## Abstract

**Background:**

Stimulation of CD40 can augment anti-cancer T cell immune responses by triggering effective activation and maturation of antigen-presenting cells (APCs). Although CD40 agonists have clinical activity in humans, the associated systemic activation of the immune system triggers dose-limiting side-effects.

**Methods:**

To increase the tumor selectivity of CD40 agonist-based therapies, we developed an approach in which soluble trimeric CD40L (sCD40L) is genetically fused to tumor targeting antibody fragments, yielding scFv:CD40L fusion proteins. We hypothesized that scFv:CD40L fusion proteins would have reduced CD40 agonist activity similar to sCD40L but will be converted to a highly agonistic membrane CD40L-like form of CD40L upon anchoring to cell surface exposed antigen via the scFv domain.

**Results:**

Targeted delivery of CD40L to the carcinoma marker EpCAM on carcinoma cells induced dose-dependent paracrine maturation of DCs ~20-fold more effective than a non-targeted control scFv:CD40L fusion protein. Similarly, targeted delivery of CD40L to the B cell leukemia marker CD20 induced effective paracrine maturation of DCs. Of note, the CD20-selective delivery of CD40L also triggered loss of cell viability in certain B cell leukemic cell lines as a result of CD20-induced apoptosis.

**Conclusions:**

Targeted delivery of CD40L to cancer cells is a promising strategy that may help to trigger cancer-localized activation of CD40 and can be modified to exert additional anti-cancer activity via the targeting domain.

## Background

The tumor necrosis factor (TNF) receptor family member CD40 is a critical regulator of cellular and humoral immunity. In line with this, CD40 is broadly expressed on immune cells, although predominantly on antigen-presenting cells (APCs) such as dendritic cells (DC) and B cells [1-3]. One of the main functions of the CD40L/CD40 system is to activate and “license” DCs to prime effective cytotoxic CD8^+^ T cell responses [4,5]. In brief, CD40 ligand (CD40L) expressed on CD4^+^ helper T cells engages CD40 on APCs and induces APC activation and maturation. In turn, such CD40-licensed APCs induce activation and proliferation of antigen-specific CD8^+^ cytotoxic T cells [6,7]. In the absence of CD40 signalling, the interaction of CD8^+^ T cells with so-called “unlicensed” APCs induces T cell anergy or triggers formation of regulatory T cells [8]. Thus, CD40 is crucial for effective generation of cytotoxic CD8^+^ T cell immune responses. Although normally induced by helper T cells, CD40 signalling on APCs can also be effectively triggered using agonistic antibodies or CD40L, thus bypassing the need for CD4^+^ T cell help [4,9]. These features delineate a clear rationale for CD40 agonist-based cancer immunotherapy.

CD40 has been explored as a target for the treatment of several forms of cancer using recombinant soluble CD40L (sCD40L) or agonistic therapeutic antibodies (Abs). In pre-clinical models, sCD40L and agonistic CD40 Abs are highly effective at inducing DC maturation and eradicating tumors (reviewed in [4]). However, an important concern for this type of immunotherapy in humans is the potential for systemic over activation of the immune system and concomitant toxicity. Indeed, dose-limiting toxicity using sCD40L or agonistic CD40 antibodies has been reported in humans [10-12]. Importantly, whereas systemic treatment with agonistic CD40 Abs in pre-clinical mouse models was associated with significant liver toxicity, local administration of agonistic CD40 Abs proved equally effective, yet without the associated toxicity [13,14].

The efficacy of CD40 signaling is dependent on the clustering of CD40 within the membrane of the targeted cells. For instance, CD40-signaling induced by soluble CD40L (sCD40L) was potentiated ~10-fold upon secondary cross-linking of CD40L into higher order multimers [15-17]. In line with this, CD40 signaling induced by anti-CD40 antibodies critically depends on the presence of Fc-receptor positive cells [18]. Based on these crosslinking requirements for CD40/CD40L signaling, CD40L has also been evaluated in a proof-of-concept study with a fibroblast activation protein (FAP)-targeted scFv:CD40L fusion protein. In brief, antibody fragment-mediated anchoring to FAP-expressing cells enabled the scFv:CD40L fusion protein to trigger IL-8 production in target cells with an ~25-fold lowered ED50 value [17].

**Figure 1:**
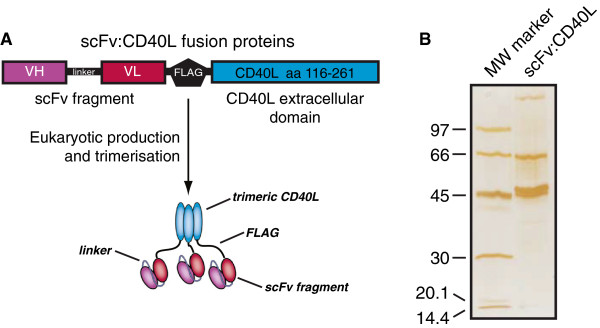
**Domain architecture and affinity purification of a scFv:CD40L fusion proteins. A** ScFv:CD40L fusion proteins comprise a single chain variable fragment (scFv) of the specificity of interest genetically linked to the extracellular domain of CD40L (amino acids 116–261) and a FLAG-tag for affinity purification. Following production in eukaryotic cells, scFv:CD40L fusion proteins form stable homotrimeric fusion proteins. **B** The purity of a scFv:CD40L fusion protein isolated by affinity chromatography was assessed by gel electrophoresis and silver staining.

Here, we further developed this targeted approach by evaluating the selective delivery of sCD40L to the well-established carcinoma marker EpCAM and the B-cell leukemia marker CD20. In brief, the resultant scFv:CD40L fusion proteins were designed to selectively deliver sCD40L to the cell surface of target antigen-positive cancer cells, thereby triggering target antigen-restricted DC maturation (see Figure 1 for schematic representation of the scFv:CD40L fusion proteins). Second, the anti-CD20 antibody fragment derived from rituximab has previously been shown to trigger CD20 cross-linking dependent apoptosis in B-cell leukemic cells in a scFv:FasL fusion protein [19]. Therefore, CD20 cross-linking by scFvCD20:CD40L may trigger apoptotic elimination of malignant B-cells. Both fusion proteins were evaluated for target cell-selective activity as determined by auto- and paracrine activation of antigen presenting cells (APCs) and scFv-mediated anti-tumor effects.

## Results

### Antibody fragment-specific binding of scFv:CD40L fusion proteins

**Figure 2:**
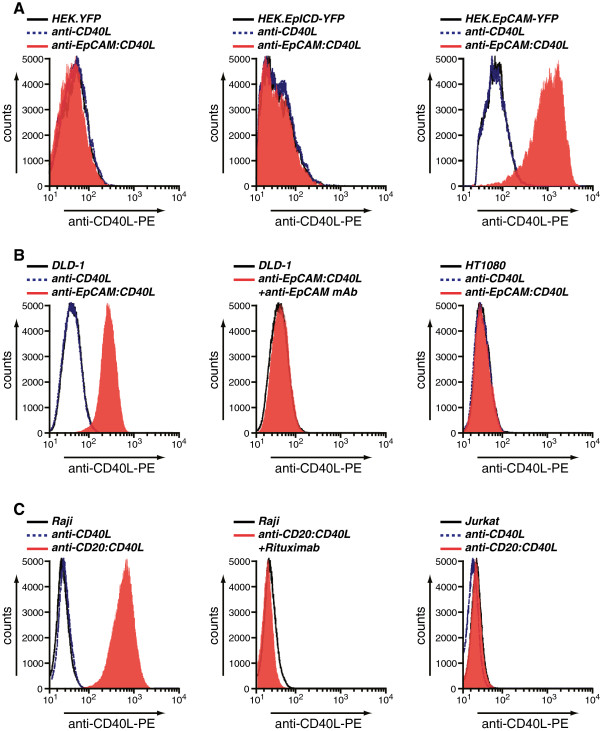
**Target antigen-restricted binding by scFv:CD40L fusion proteins. A** HEK.YFP, HEK.EpICD-YFP, HEK.EpCAM-YFP or **B** DLD-1 and HT1080 cells were incubated with anti-EpCAM:CD40L for 1 h, washed 3 times with PBS and binding assessed by flow cytometry using a PE-conjugated anti-CD40L antibody. To determined specific binding, cells were pretreated with parental anti-EpCAM antibody as indicated. **C** Raji B cells or Jurkat T cells were incubated with anti-CD20:CD40L for 1 h, washed 3 times with PBS and binding assessed by flow cytometry using a PE-conjugated anti-CD40L antibody. To determined specific binding, cells were pretreated with parental anti-CD20 antibody rituximab as indicated.

To determine the binding characteristics of anti-EpCAM:CD40L, the EpCAM-negative cell line HEK293 and the EpCAM-transfectant cell line HEK293.EpCAM were incubated with anti-EpCAM:CD40L and analyzed for CD40L by flow cytometry. Incubation of EpCAM-negative HEK293 cells with anti-EpCAM:CD40L did not trigger an increase in fluorescence upon anti-CD40L antibody staining (Figure 2A; left panel). By contrast, incubation of HEK293 cells engineered to express EpCAM (HEK.EpCAM) with anti-EpCAM:CD40L resulted in a significant increase in surface CD40L staining (Figure 2A; right panel). As with HEK293, anti-EpCAM:CD40L did not bind HEK293 cells that only expressed the intracellular domain of EpCAM (HEK293.EpICD; Figure 2A; middle panel). Further, incubation of the EpCAM^+^ colon carcinoma cell line DLD-1 with anti-EpCAM:CD40L showed a strong anti-CD40L fluorescence signal (Figure 2B; left panel) that was completely inhibited by pre-incubation with an epitope-competing EpCAM-blocking antibody (Figure 2B, middle panel). Similar antibody fragment-selective binding characteristics were obtained for the anti-CD20:CD40L fusion protein, with selective binding to CD20^+^ B cells (Figure 2C; left panel) that could be inhibited with the parental mAb rituximab (Figure 2C; middle panel). Furthermore, anti-CD20:CD40L failed to bind to CD20^-^ Jurkat T cells (Figure 2C; right panel). Taken together, these data demonstrate that the scFv:CD40L fusion proteins have a high binding affinity for their respective target antigens EpCAM and CD20.

### CD40L remains biologically active within scFv:CD40L fusion proteins

**Figure 3:**
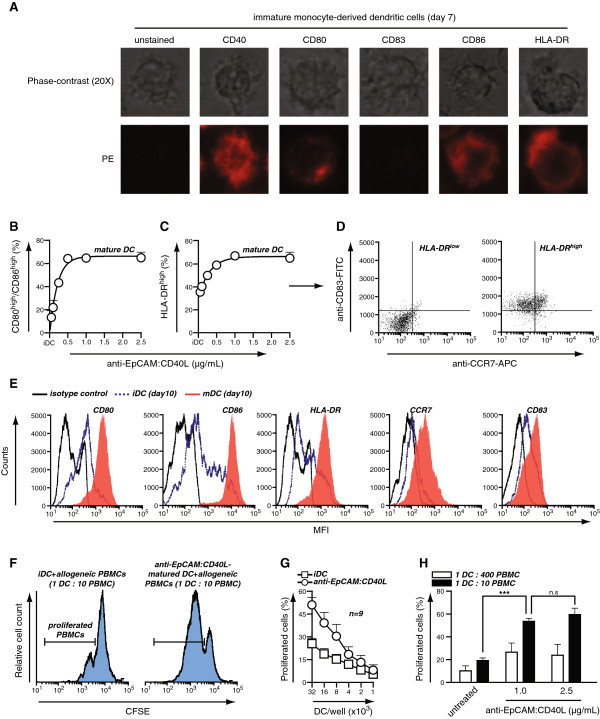
**CD40L remains biologically active in the context of scFv:CD40L fusion proteins. A** Phenotypical and morphological characterization of monocyte-derived DC (moDC) used for the study. After induction, moDC were harvested, stained with PE-conjugated antibodies specific for CD40, CD80, CD83, CD86 and HLA-DR, and expression of the indicated molecules assessed using fluorescent microscopy. **B-D** Immature moDC were treated for 3 days as indicated and cells with high levels of co-expressed CD80 and CD86 quantified using flow cytometry **(B)**. In parallel, the expression of CD83 and CCR7 was assessed in HLA-DR^high^ and HLA-DR^low^ cells **(C-D)**. **E** Representative flow cytometric plots of CD80, CD86, HLA-DR, CCR7 and CD83 expression on moDC matured for 3 days with 1 μg/mL anti-EpCAM:CD40L. **F** iDC and anti-EpCAM:CD40L-matured DC (1 μg/mL) were co-cultured with 1x10^5^ CFSE-labeled allogeneic CD3^+^ T cells at the indicated cell numbers. T cell proliferation was determined by assessing loss of CFSE. **G** iDC were matured using the indicated concentrations of anti-EpCAM:CD40L and allogeneic T cell proliferation assessed as described. **H** Representative CFSE profiles of the experiment performed in G.

To verify that N-terminal fusion of an scFv antibody fragment to sCD40L did not negatively affect the biological activity of the trimeric sCD40L, the capacity of scFv:CD40L fusion proteins to induce DC-maturation was assessed using anti-EpCAM:CD40L. Of note, non-targeted sCD40L has previously been shown to induce DC-maturation at relatively high concentrations ranging from 1 to 3 μg/ml [20]. Therefore, monocyte-derived immature dendritic cells (iDCs) were treated with high concentrations of the scFv:CD40L proteins (1 μg/ml) and analyzed for induction of DC maturation markers (Additional file 1: Figure S1A for protocol). Monocyte-derived iDCs had a typical DC morphology and expressed low cell surface levels of CD80 and CD86, intermediate levels of CD40 and HLA-DR, and lacked expression of CD83, a marker for mature DCs (mDC) (Figure 3A). Treatment of iDC with anti-EpCAM:CD40L dose-dependently increased the percentage of CD80^high^/CD86^high^ DCs (Figure 3B) and significantly up-regulated HLA-DR expression (Figure 3C). Importantly, the HLA-DR^high^ but not the HLA-DR^low^ DCs also expressed the mDC markers CD83 and CCR7 (Figure 3D; histograms and MFI for all markers in Figure 3E and Additional file 1: Figure S1B, respectively) and retained a CD14-negative phenotype (Additional file 1: Figure S1C). These changes were in line with the phenotypic changes observed after treatment of iDC with lipopolysacharide (LPS), a well-established strong trigger of DC maturation (Additional file 1: Figure S1D). In line with this maturation marker profile, anti-EpCAM:CD40L induced significant production of the pro-inflammatory DC cytokine IL-12 after 3 days, whereas untreated iDC produced negligible levels of IL-12 (Additional file 1: Figure S1E).

Next, the anti-EpCAM:CD40L-matured DCs were analyzed for their capacity to induce T cell proliferation in allogeneic PBMCs. Hereto, allogeneic PBMCs were labeled with CFSE and mixed with DCs at the indicated ratios, whereupon T-cell proliferation in mixed cultures of iDC/PBMC and anti-EpCAM:CD40L matured DCs/PBMCs was analyzed after 7 days. In these mixed cultures, anti-EpCAM:CD40L matured DCs proved more potent at inducing T cell proliferation than iDC (exemplary proliferation plots in Figure 3F, quantified in Figure 3G), with a significant increase in the percentage of proliferating T cells (Figure 3H). Taken together, these data demonstrated that the soluble CD40L domain in scFv:CD40L fusion proteins remains biologically active and, in correspondence with its mode of action as a soluble molecule, requires high concentrations over prolonged periods of time to trigger efficient CD40 responses.

### Cell surface-immobilization of CD40L on cancer cells augments paracrine maturation of iDC

**Figure 4:**
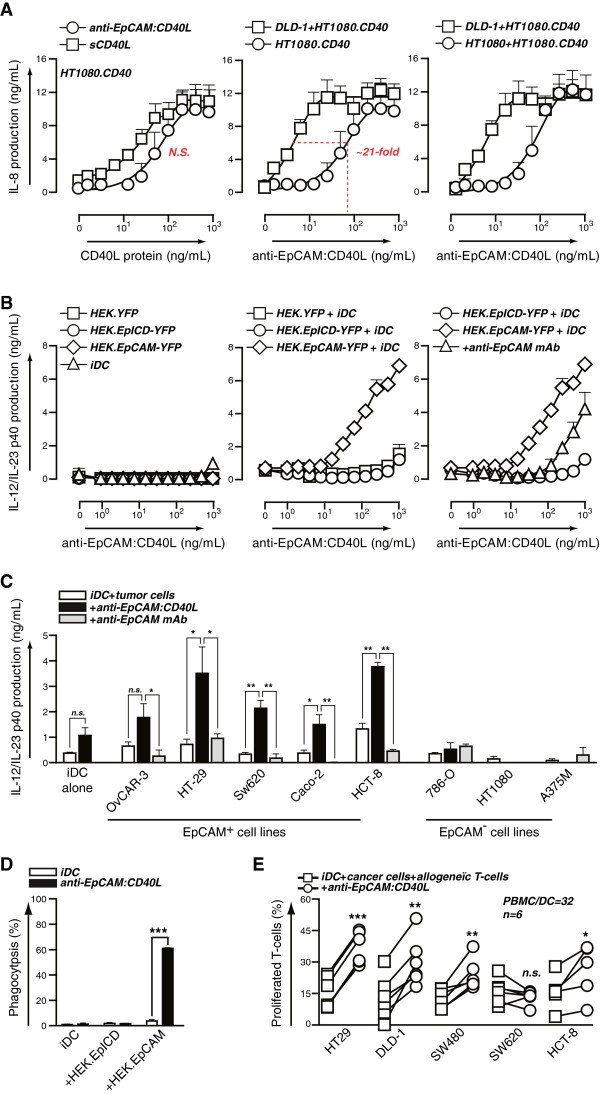
**EpCAM-restricted paracrine maturation of immature DC by anti-EpCAM:CD40L. A** Indicated mono- and/or co-cultures of HT1080, HT1080.CD40 and DLD-1 were treated with increasing concentrations of anti-EpCAM:CD40L and CD40 signaling assessed by measuring production of IL-8. **B** Indicated mono- and co-cultures of HEK.YFP, HEK.EpICD-YFP, HEK.EpCAM-YFP and immature moDC (iDC) were treated overnight with increasing concentrations of anti-EpCAM:CD40L. iDC maturation was assessed by determining the concentration of IL-12/23 in the culture supernatant. To determine antigen-dependent effects, cells were pre-treated with parental anti-EpCAM blocking antibody. **C** iDC were co-cultured overnight with the indicated tumor cell lines in the presence or absence of 100 ng/mL anti-EpCAM:CD40L ± anti-EpCAM blocking antibody. iDC maturation was assessed by determining the concentration of IL-12/23 in the culture supernatant. **D** HEK293.YFP, HEK293.EpICD-YFP or HEK293.EpCAM-YFP cells were co-cultured for 2 h with iDC at a 1:1 ratio in the presence or absence of anti-EpCAM:CD40L. Phagocytosis by the CD80^high^/CD86^high^ population was determined using flow cytometry. **E** iDC were co-cultured for 3 days with the indicated tumor cell lines in the presence or absence of anti-EpCAM:CD40L. Co-cultures were harvested, inactivated and replated at the indicated ratio with PBMCs. T cell proliferation was assessed 6 days later as described.

To evaluate whether target antigen-selective immobilization of scFv:CD40L would augment CD40 signaling, EpCAM^-^CD40^-^ HT1080 cells and CD40-transfected HT1080 cells (HT1080.CD40) were co-cultured with EpCAM^+^/CD40^-^ DLD-1. Subsequently, induction of CD40 signaling was analyzed by measuring IL-8 production. Treatment of HT1080.CD40 with anti-EpCAM:CD40L triggered IL-8 at higher doses of 100–1000 ng/ml, in line with the activity profile of sCD40L (Figure 4A; left panel). However, in co-cultures of HT1080.CD40 with EpCAM^+^ DLD-1 cells, the treatment with anti-EpCAM:CD40L resulted in IL-8 secretion at significantly lower doses, corresponding to a ~20-fold down-shift in the ED50 value (Figure 4A; middle panel). In mixed cultures of parental HT1080 with DLD-1 cells, no IL-8 secretion was detected (Additional file 1: Figure S1F).

To subsequently assess the potentiating effect of targeted delivery of CD40L on DC maturation, monocyte-derived iDC were co-cultured with HEK293.EpCAM and overnight production of IL-12 was determined as read-out for iDC maturation. Monocultures of HEK293.EpCAM and iDCs treated with increasing concentrations of anti-EpCAM:CD40L did not produce IL-12 even at 1 μg/mL (Figure 4B: left panel). By contrast, in iDC/HEK.EpCAM co-cultures, treatment with anti-EpCAM:CD40L dose-dependently induced IL-12 production (Figure 4B: middle panel). IL-12 production was dependent on EpCAM-specific binding since pre-incubation with the parental EpCAM blocking antibody largely blocked IL-12 production (Figure 4B: right panel). In control mixed cultures of iDC with HEK.YFP and HEK.EpICD only marginal levels of IL-12 were detectable (Figure 4B: middle panel). Furthermore, in co-culture experiments of iDCs with a panel of EpCAM^+^ cancer cell lines, treatment with anti-EpCAM:CD40L induced significant IL-12 production that was inhibited by the EpCAM blocking antibody (Figure 4C). By contrast, no IL-12 was detected upon treatment of co-cultures of iDCs and EpCAM^-^ cancer cell lines with anti-EpCAM:CD40L (Figure 4C), nor when EpCAM^+^ cells were treated with an scFv:CD40L fusion protein of irrelevant specificity (Additional file 1: Figure S1G).

In line with this IL-12 production, treatment of co-cultures of iDC and HEK.EpCAM-YFP with anti-EpCAM:CD40L also triggered phagocytic uptake of high numbers of HEK.EpCAM cells (Figure 4D), whereas no such phagocytic uptake was detected in co-cultures with HEK or HEK.EpICD cells (Figure 4D). Next, we assessed whether these findings extended to DC-induced T cell proliferation. To this end, we co-cultured a panel of five EpCAM^+^ cancer cell lines with iDCs and allogeneic T cells in the presence or absence of anti-EpCAM:CD40L using a modified procedure of the proliferation assay described above. Importantly, in 4 out of 5 cells lines, treatment with anti-EpCAM:CD40L was associated with increased T cell proliferation (Figure 4E).

### Targeted delivery of CD40L to CD20 induces paracrine maturation of dendritic cells

**Figure 5:**
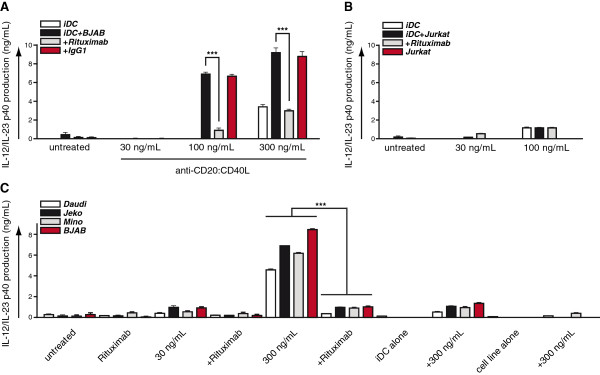
**CD20-restricted paracrine maturation of immature DC by anti-CD20:CD40L. A** iDC were co-cultured overnight with BJAB in the presence or absence of either 30, 100 or 300 ng/mL anti-CD20:CD40L ± parental antibody Rituximab or irrelevant isotype control. iDC maturation was assessed by determining the concentration of IL-12/23 in the culture supernatant. **B** Indicated mono- and co-cultures of iDC and Jurkat T leukemia cells were treated overnight with 30 or 300 ng/mL anti-CD20:CD40L ± Rituximab. iDC maturation was assessed by determining the concentration of IL-12/23 in the culture supernatant. **C** iDC were co-cultured overnight with the indicated tumor cell lines in the presence or absence of 30 or 300 ng/mL anti-CD20:CD40L ± Rituximab. iDC maturation was assessed by determining the concentration of IL-12/23 in the culture supernatant.

To assess whether these effects could be extended to leukemic cancer cells, we next investigated the anti-CD20:CD40L fusion protein. Similar to the effects observed for anti-EpCAM:CD40L, monocultures of iDCs treated with increasing concentrations of anti-CD20:CD40L only produced marginal levels of IL-12 (Figure 5A). By contrast, in co-cultures of iDC with the CD20^+^ cell line BJAB, treatment with anti-CD20:CD40L dose-dependently induced IL-12 production (Figure 5A). IL-12 production was dependent on CD20-binding since pre-incubation with the parental CD20 blocking antibody rituximab fully abrogated the increased IL-12 production (Figure 5A). In control mixed cultures of iDC with CD20^-^ Jurkat stimulated with anti-CD20:CD40L, only marginal levels of IL-12 were detected (Figure 5B). These findings were extended to the B cell leukemic lines Mino, Jeko and Daudi with BJAB serving as positive control (Figure 5C). Treatment of iDC:B cell co-cultures with either 30 or 300 ng/mL anti-CD20:CD40L induced dose-dependent production of IL-12 across all cell lines. As before, rituximab fully abrogated the induction of IL-12, indicating a CD20-dependent effect. Of note, treatment of B cell lines or iDC alone induced no or only marginal production of IL-12 in these experiments (Figure 5C).

### Targeted delivery of CD40L to leukemic cell lines induces non-proliferative CD40 signaling

In leukemic cells, signaling via CD40 has been linked to both pro-survival and anti-cancer effects [21,22]. However, most of these studies have been performed using varying CD40-targeted agents (e.g. crosslinked CD40L or antibodies), complicating interpretation. Therefore, to assess the direct effects of anti-CD20:CD40L on leukemic B cells, BJAB and Raji were treated for 3 days with increasing concentrations of anti-CD20:CD40L and changes in cell surface expression of B cell maturation markers were assessed. In these experiments, the optimal dose for non-saturating induction of signaling was determined at 250 ng/mL (not shown). In both cell lines, treatment with anti-CD20:CD40L induced an increase in cell surface CCR7 (Additional file 1: Figure S1H and S1I), whereas no significant changes were observed in the cell surface levels of HLA-DR, CD80 or CD86 (Additional file 1: Figure S1H and S1I). Interestingly, treatment with anti-CD20:CD40L induced an upregulation of CD83 on BJAB cells and a downregulation of CD83 on Raji cells, indicating cell type-specific effects of CD40 signaling (Additional file 1: Figure S1H and S1I). As expected, treatment of leukemic B cell lines with control fusion protein anti-EpCAM:CD40L had no effect in this setting even when applied at concentrations up to 1 μg/mL (not shown). Finally, we did not observe proliferation or cell death as a result of treatment with anti-CD20:CD40L within this time frame (not shown).

### scFv-mediated delivery of CD40L induces secondary scFv-dependent effects in cancer cells

**Figure 6:**
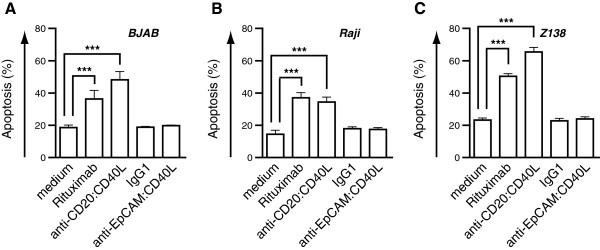
**scFv-dependent signaling by scFv:CD40L fusion proteins. A** BJAB, **B** Raji and **C** Z138 B leukemia cell lines were treated for 5 days with 1 μg/mL Rituximab, anti-CD20:CD40L, irrelevant IgG1 control antibody, or anti-EpCAM:CD40L and cell death assessed by loss of mitochondrial membrane potential.

We previously demonstrated a synergistic anti-cancer effect by an anti-CD20 fusion protein that relied on simultaneous induction of 1. death receptor-mediated apoptosis by FasL and 2. cell death via crosslinking of CD20 by the Rituximab scFv [19]. Therefore, we assessed whether targeted delivery of CD40L to CD20 by means of anti-CD20:CD40L could likewise induce CD20-mediated cell death. Treatment of leukemic B cell lines BJAB, Raji and Z138 induced a significant loss of viable cells after 5d, similar to that observed for treatment with rituximab (Figure 6A-C). Treatment of cells with anti-EpCAM:CD40L had no apparent effects at concentrations up to 1 μg/mL (Figure 6A-C). Of note, induction of cell death by rituximab occurred only in the presence of a secondary crosslinker, whereas anti-CD20:CD40L-induced cell death did not require oligomerization. These findings are in agreement with the data on anti-EpCAM:CD40L and suggest a model where binding of anti-CD20:CD40L to the cell surface induces CD40-clustering and concomitant crosslinking of CD20.

## Discussion

We explored targeted delivery of CD40 ligand to the cancer cell-surface as a therapeutic option for treatment of malignancies. To this end, we generated EpCAM- and CD20-targeted fusion proteins, anti-EpCAM:CD40L and anti-CD20:CD40L, respectively, and analyzed functional immune parameters. Based on our findings, CD40L remains biologically active in scFv fusion proteins, with an activity profile similar to soluble CD40L. However, binding of CD40L fusion proteins to target antigens on the cancer cell surface promotes oligomerization and augmented activation of CD40 on neighboring immature DC. As a result, maturation of iDC occurred at approximately 20 fold lower concentrations as in the case of stimulation with “unbound” CD40L. In addition, we describe how targeted delivery of CD40L to CD20^+^ leukemic B cells can induce simultaneous B cell death and paracrine maturation of iDC. Importantly, B cell lines did not undergo additional proliferation as a result of treatment with anti-CD20:CD40L. These findings are in agreement with earlier reports on therapeutic exploitation of CD40 and highlight our approach as a means of achieving loco-regional maturation of DC in the tumor micro-environment [13]. Furthermore, rational incorporation of scFv fragments into these fusion proteins can be used to confer additional anti-cancer effects.

Our data on the cell surface anchoring requirements of soluble CD40L for optimal induction of CD40 signaling highlight the importance of choosing the right preparation of TNF family ligands for therapeutic exploitation. Typically, ligands of the TNF family are transmembrane proteins of which a significant fraction can be cleaved to yield soluble molecules [23-25]. In general, these soluble molecules have a reduced receptor activating potential that can be (partially) restored upon artificial (antibody-mediated) crosslinking or by artificial cell surface anchoring. While constitutively inducing ligand oligomerization to optimize signaling activity could promote off-target systemic toxicity, cell surface immobilization can “open” the therapeutic window.

This might also be of relevance for therapies that base on CD40 activation. Both pre-clinical and clinical studies have highlighted toxicity concerns with the use of agonistic CD40-targeted agents for treating cancer [10,11,26]. Indeed, in the first clinical trials with the agonistic anti-CD40 antibody CP-870,893, grade 1 to 2 toxicities in the form of cytokine release syndrome were observed in ~55% of patients and two grade 3 toxicities out of 27 patients were reported. Nevertheless, 4 out of 27 patients had a partial response indicating the potential of targeting CD40 [10]. Similarly, 2 out of 32 patients administered soluble recombinant human CD40L (rhCD40L) displayed signs of a partial response, although treatment was associated with transient elevations of serum liver transaminases (grade 3 or 4 severity) in 28% of the patients treated with the maximum tolerated dose (MTD; of 0.1 mg/kg) [27]. Of note, these transient signs of liver toxicity have also frequently been reported in preclinical work on CD40-targeted therapeutics (discussed in [13].

To overcome these toxicity issues in the preclinical setting, the group of Melief et al. has previously evaluated a local release strategy for immunomodulatory antibodies [14]. In their approach, an anti-CD40 antibody is dissolved in a slow release formulation of the adjuvant Montanide and injected near the tumor site. After this loco-regional application, the antibody is proposed to drain into the lymphatic system and (re-)activate resident DCs to mount an anti-tumor immune response. Since DC in the draining nodes are exposed to tumor associated antigens, a systemic immune response is induced that could eradicate contra lateral tumor masses in these animals. Importantly, this approach was associated with significantly reduced liver toxicity compared to systemic injection, and allowed for much higher local dosage. Nevertheless, loco regional application remains unfeasible for most cancers.

**Figure 7:**
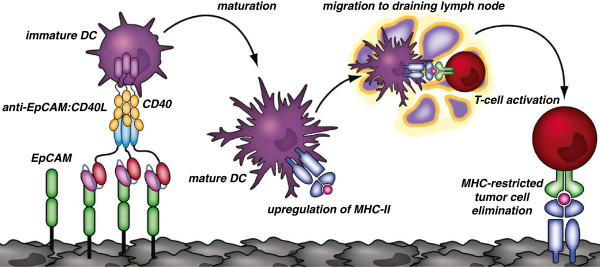
**Schematic representation of the proposed mode of action for scFv:CD40L fusion proteins; exemplified for anti-EpCAM:CD40L.** Binding of anti-EpCAM:CD40L to EpCAM^+^ target cancer cells induces paracrine CD40 signaling in tumor resident immature dendritic cells (iDC). Subsequent maturation of iDC results in migration to draining lymph nodes and upregulation of antigen-presenting and co-stimulatory molecules. Mature DC (mDC) can subsequently induce specific T cell activation and anti-tumor immunity.

In our targeted approach, we expect to achieve a similar therapeutic efficacy as local release with the advantage of systemic application (Figure 7). As mentioned, the scFv:CD40L is anticipated to be largely inactive “en route” to the cancer cells by virtue of 1. the higher affinity of the scFv for the tumor antigen compared to CD40L for CD40 and 2. due to the limited signalling activity of soluble CD40L. Indeed, binding of CD40L to CD40 in our flow cytometric experiments was weaker compared to antigen-mediated binding. To reduce off target effects, rational selection of a target antigen is therefore a key factor in design of such therapeutics. Here, we chose EpCAM to target CD40L to the cell surface of epithelial cancer. In normal epithelia, EpCAM is thought to be shielded by an intact basal lamina, thereby preventing binding and accumulation of the anti-EpCAM:CD40L fusion protein on untransformed cells [28-30]. During malignant transformation, polarity is lost and EpCAM exposed to antibody-based therapeutics.

For targeting B cells, we chose the antigen CD20. CD20 is expressed on both healthy and malignant B cells and is a well-established target for therapy of B cell leukaemia (i.e. Rituximab) [31,32]. CD20-targeted therapeutics are generally well tolerated and transient depletion of healthy B cells can be clinically managed [31,32]. However, care should be taken with targeted delivery of CD40L for treatment of B cell malignancies as several studies have demonstrated a role for CD40 in promoting progression of malignancies, and antagonistic anti-CD40 antibodies have met with clinical success in the treatment of certain haematological cancers [21,22,33]. Nevertheless, in line with earlier findings we observed no growth or survival promoting effects of anti-CD20:CD40L on leukemic B cells at doses up to 1 μg/mL and/or extended 5 day treatment. However, we cannot exclude the possibility that concurrent signalling via CD20 abrogates the proliferative aspects that are normally observed with activation of CD40. Indeed, crosslinking of CD20 via the rituximab-derived anti-CD20 scFv induced a potent loss of cell viability in a subset of leukemic B cells.

## Conclusions

We describe how targeted delivery of CD40L to cancer antigens can be exploited to achieve localized activation of CD40 and can be modified to exert additional anti-cancer action via the targeting domain. To our knowledge, this is first report of antigen-restricted maturation of antigen presenting cells by CD40L. Further studies should aim to elucidate whether this approach can be used to improve the anti-tumor efficacy and safety profile of CD40 activation *in vivo*.

## Materials & methods

### Plasmids

Expression vectors encoding the Flag-tagged CD40 ligand are derived from a variant of pCR3 (Invitrogen), which was provided by P. Schneider (University of Lausanne, Epalinges, Switzerland) and encodes an Ig signal peptide followed by a Flag tag. To obtain scFvRit:CD40L (anti-CD20:CD40L), amplicons of CD40L encoding amino acids 116–261 including a stop codon were first inserted in frame 5’ to the Flag tag by standard cloning techniques. Next, the leader encoding fragment of the Flag-ligand encoding plasmid was replaced by an amplicon encoding the leader sequence plus the scFvRit cDNA as previously reported [17]. scFvC54 (anti-EpCAM) was generated analogously using PCR from vector pEE14-scFvC54:TRAIL and inserted 5’ to the Flag tag as described above.

### Production and purification

ScFvRit:CD40L (anti-CD20:CD40L) and scFvC54:CD40L (anti-EpCAM:CD40L) were produced in HEK293 cells by transient transfection of the expression vector. In brief, cells were electroporated (50 × 10^6^ cells/ml, 4-mm cuvette, 250 V, 1800 μF, maximum resistance) with 30 μg of DNA in 1 ml of culture medium containing 10% FCS using an Easyject Plus electroporator (PeqLab). Electroporated cells were recovered overnight in RPMI 1640 with 10% FCS (PAA Laboratories), and the next day the medium was replaced by low serum RPMI 1640 (0.5% FCS). After 3–4 days supernatants were collected and clarified by centrifugation. Fusion proteins were purified by affinity chromatography on anti-Flag M2 agarose beads (Sigma-Aldrich) and eluted with TBS containing 100 μg/ml Flag peptide (Sigma-Aldrich). The fractions containing the recombinant proteins were finally dialyzed against PBS, purity assessed by gel electrophoresis (Figure 1B) and stored at -20°C for further analysis.

### Cell lines

OvCAR-3, HT-29, DLD-1, HCT-8, Sw480, Sw620, Caco-2, HT1080, HEK293, A375M, 786-O, BJAB, Jurkat, Daudi, Jeko and Mino were obtained from the American Tissue Culture Collection (ATCC) and characterized by short tandem repeat profiling, karyotyping and isoenzyme analysis. HT1080.CD40 cells were generated as described before. HEK293.YFP, HEK293.EpICD-YFP and HEK.EpCAM-YFP were a kind gift from Dr. O. Gires (University of München) [34]. Cells were cultured in RPMI 1640 medium supplemented with 10% fetal calf serum and 500 μg/ml Geneticin.

### Isolation, cultivation, and stimulation of primary cells

Monocytes and PBMCs were isolated from rests of blood buffy coats of fully anonymized donors obtained from the Institute of Clinical Transfusion Medicine and Hämotherapy of the University Hospital Würzburg and required no special written informed consent. PBMCs were isolated by density gradient centrifugation with lymphocyte separation medium (PAA Laboratories) [35]. Monocytes were separated from PBMCs by MACS magnetic bead separation with anti-CD14-coated beads (Miltenyi Biotec). Monocytes were seeded in 6-well plates (3 × 10^6^ cells) with RPMI 1640, 10% FCS, and penicillin/streptomycin. For DC differentiation 100 ng/ml GM-CSF and 20 ng/ml IL-4 (ImmunoTools) were added every second day. On day 7, cells were stimulated as indicated. On day 10, functional analyses were performed as indicated.

### FACS analysis and fluorescent microscopy

To determine cell surface expression of the indicated markers by flow cytometry, cells were incubated on ice with anti-CD14-FITC, anti-CD80-PE, anti-CD83-FITC, anti-CD86-APC, anti-CCR7-APC, anti-HLA-DR-PE and/or indicated combinations thereof or the appropriate isotype control Abs (all from Becton Dickinson, Amsterdam, The Netherlands). After three washes with ice-cold PBS, cell-associated immunofluorescence was determined by FACS analysis. For binding studies with the scFv:CD40L constructs, indicated cell lines were incubated on ice for 1 h with scFv:CD40L fusion proteins, washed three times with ice-cold PBS and stained with anti-CD40L-PE antibody (Becton Dickinson, Amsterdam, The Netherlands) on ice for another 1 h. Subsequently, cells were washed three more times with ice-cold PBS and used for flow cytometry. For blocking studies, cells were pre-incubated with control antibodies (Rituximab or MOC31-Fc) for 1 h on ice prior to incubation with fusion protein.

For immunofluorescence, cells were washed and stained with the indicated PE-conjugated antibodies (anti-CD40-PE, anti-CD80-PE, anti-CD83-PE, anti-CD86-PE and anti-HLA-DR-PE all from Becton Dickinson, Amsterdam, The Netherlands) on ice. After three washes with ice-cold PBS, cell-associated immunofluorescence was determined by fluorescent microscopy. All fluorescent microscopy was performed on an EVOS fl Digital Fluorescence Microscope.

### ELISA analysis

Cancer cells were seeded (2 × 10^4^ cells/well) in 96-well tissue cultures plates in 100 μl of RPMI 1640 medium with 10% FCS and grown overnight. On the next day, medium was exchanged to minimize the background of constitutive cytokine production, and cells were stimulated for 16 h with the indicated concentrations of fusion protein ± blocking antibody in the presence or absence of indicated HT1080 transfectants or Dendritic cells. Supernatants were analyzed for production of IL-8 or IL-12 using the BD human IL-8 ELISA or IL-12/IL-23 Duoset ELISA (BD Biosciences) according to the manufacturer’s instructions.

### T cell proliferation

Dendritic cells (DCs) matured as indicated (or relevant controls) were harvested, washed three times and resuspended at a density of 1×10^7^ cells/mL. DCs were subsequently incubated with 50 μg/mL Mitomycin C solution in PBS (freshly prepared and filter sterilized at 0.5 mg/mL) in the dark for 20 min. at 37°C. After incubation, Mitomycin C was rapidly diluted by addition of an excess of warm culture medium and cells were washed three more times by centrifugation. Cells were resuspended at a density of 10×10^6^ cells/mL and plated at the indicated densities/ratios in round-bottom 96-well plates in the presence of 1×10^5^ T cells/well. DC-T cell co-cultures were briefly centrifuged (1 min, 100 g) to promote cell-cell contact, incubated for 6 days at 37°C in a humidified 5% CO_2_ incubator and T cell proliferation assessed by CFSE dilution on a flow cytometer. For CFSE-labeling, the Vybrant^®^ CFDA SE Cell Tracer Kit (Invitrogen) was used according to the manufacturer’s instructions. In all experiments, T cells autologous to the Dendritic cell population were used as control and never displayed >5% proliferation. For co-cultures of dendritic cells and tumor cells this procedure was slightly modified. First, the ratios used in co-culture of immature dendritic cells and tumor cells were optimized per cell line to prevent cancer cells from overgrowing. Second, the percentage of viable dendritic cells within the mixed population after 3 day maturation was assessed using CD86/CD83/PI staining on flow cytometry and cell counts adjusted accordingly to a PBMC/DC ratio of 32. Of note, mitomycin C treatment did not fully abrogate cancer cell growth during the 6 day MLR, but slowly proliferating cancer cells did not affect T cell viability within this time frame.

### Phagocytosis

HEK293.YFP, HEK293.EpICD-YFP or HEK293.EpCAM-YFP cells were harvested and kept in suspension. Cells were added at a 1:1 ratio to cultures of iDC in the presence or absence of anti-EpCAM:CD40L for 2 h, washed three times with ice cold PBS and stained with anti-CD80 and anti-CD86 antibodies as described in the section on flow cytometry. Cells were gated exclusively on the single cell population by FSC/SSC analysis to exclude DC:HEK cell doublets. DCs were identified based on co-expression of CD80 and CD86 expression and the increase in YFP signal assessed in the FL1 channel by means of the indicated histograms. Percentages were determined based on the amount of YFP^+^ cells within the total CD80/CD86 population.

### B cell maturation and cell death

Primary B cells were cultured for up to 5d as indicated in 6-well plates (1×10^6^ cells/well; 3 mL/well) in the presence or absence of the indicated concentrations of anti-CD20:CD40L and harvested for analysis. To determine specific maturation, binding of anti-CD20:CD40L was blocked by pre- and subsequently co-incubating cells with 10 μg/mL of parental antibody rituximab. Where indicated, B cells were also treated with (Fab-fragment crosslinked) Rituximab, an irrelevant IgG1 control, or anti-EpCAM:CD40L, all at a final concentration of 1 μg/mL. B cell maturation was assessed by flow cytometry as described above. Cell death in B cell lines was evaluated by loss of mitochondrial membrane potential (ΔΨ) using DioC6 according to the manufacturer’s instructions (Molecular Probes).

### Statistical analysis

Data reported are mean values ± SD of at least three independent experiments. Statistical analysis was performed by one-way ANOVA followed by Tukey-Kramer post test or, where appropriate, by two-sided unpaired Student’s *t*-test. p < 0.05 was defined as a statistically significant difference. Where indicated * = p < 0.05; ** = p < 0.01; *** = p < 0.001.

## Competing interests

The authors report no conflicts of interest.

## Authors’ contributions

MB, HW, HN, DS and GG designed research. MB, KB, CS, AR and MG performed research. MB, KB and HW analyzed data. MB, HN, WH, HW and EB wrote the paper. All authors read and approved the final manuscript.

## Supplementary Material

Additional file 1: Figure S1 Schematic representation of the proposed mode of action for scFv:CD40L fusion proteins; exemplified for anti-EpCAM:CD40L. A Protocol for DC maturation and T cell proliferation used in this study. B Mean fluorescent intensities (MFI) for the DC maturation markers CD80, CD86, HLA-DR, CCR7, CD84 and CD40 as depicted in Figure 3C-E were quantified from 4 independent experiments with different donors. C After induction, immature moDC (iDC) and mature moDC (mDC) were harvested, stained with FITC-conjugated antibody specific for CD14 and expression assessed using flow cytometry. D iDC were treated for 72 h with 1 μg/mL anti-EpCAM:CD40L or 1 μg/mL Lipopolysaccharide (LPS) and the percentage of DC expressing CD86, CD83 or HLA-DR quantified by flow cytometry (n = 3). E iDC were treated for 72 h with 1 μg/mL anti-EpCAM:CD40L and production of IL-12/23 assessed by ELISA on the culture supernatant. F Indicated mono- and co-cultures of HT1080, HT1080.CD40 and DLD-1 were treated overnight with increasing concentrations of anti-EpCAM:CD40L. CD40 signaling was assessed by determining the concentration of IL-8 in the culture supernatant. G iDC were co-cultured overnight with HEK.EpCAM-YFP cells and treated with increasing concentrations of anti-EpCAM:CD40L or anti-CD20:CD40L. iDC maturation was assessed by determining the concentration of IL-12/23 in the culture supernatant. H BJAB and I Raji B cell leukemia lines were treated for 5d with anti-CD20:CD40L and maturation assessed by flow cytometry using fluorescently-conjugated anti-CD80, CD86, HLA-DR, CCR7 and CD83 specific antibodies. Click here for file
